# Management of Non-Vitamin K Antagonist Oral Anticoagulants in the Perioperative Setting

**DOI:** 10.1155/2014/385014

**Published:** 2014-09-03

**Authors:** Anne-Sophie Dincq, Sarah Lessire, Jonathan Douxfils, Jean-Michel Dogné, Maximilien Gourdin, François Mullier

**Affiliations:** ^1^Department of Anesthesiology, CHU Dinant Godinne UcL Namur, Namur Thrombosis and Hemostasis Center (NTHC), Namur Research Institute of Life Sciences (NARILIS), 5530 Yvoir, Belgium; ^2^Department of Pharmacy, University of Namur, Namur Thrombosis and Hemostasis Center (NTHC), Namur Research Institute of Life Sciences (NARILIS), 5000 Namur, Belgium; ^3^Haematology Laboratory, CHU Dinant Godinne UcL Namur, Namur Thrombosis and Hemostasis Center (NTHC), Namur Research Institute of Life Sciences (NARILIS), 5530 Yvoir, Belgium

## Abstract

The field of oral anticoagulation has evolved with the arrival of non-vitamin K antagonist oral anticoagulants (NOACs) including an anti-IIa agent (dabigatran etexilate) and anti-Xa agents (rivaroxaban and apixaban). The main specificities of these drugs are predictable pharmacokinetics and pharmacodynamics but special attention should be paid in the elderly, in case of renal dysfunction and in case of emergency. In addition, their perioperative management is challenging, especially with the absence of specific antidotes. Effectively, periods of interruption before surgery or invasive procedures depend on half-life and keeping a permanent balance between bleeding and thromboembolic risks. In addition, few data regarding the link between plasma concentrations and their effects are provided. Routine laboratory tests are altered by NOACs and quantitative measurements are not widely performed. This paper provides a review on the management of NOACs in the perioperative setting, including the estimation of the bleeding and thrombotic risk, the periods of interruption, the indication of heparin bridging, the usefulness of laboratory tests before surgery or invasive procedure, and the time of resuming. Most data are based on expert's opinions.

## 1. Introduction

Three non-vitamin K antagonist oral anticoagulants (NOACs) [1] are already widely used in the clinical setting: rivaroxaban and apixaban, two direct factor Xa (FXa) inhibitors, and dabigatran etexilate (DE)—the prodrug of dabigatran, a direct thrombin inhibitor. Both of these drugs will progressively tend to replace vitamin K antagonists (VKAs) in most of their indications. NOACs indications vary among countries. They are licensed for long-term prevention of thromboembolic events in nonvalvular atrial fibrillation (NVAF), for thromboprophylaxis of venous thromboembolism (VTE) including deep venous thromboembolism (DVT) and pulmonary embolism (PE) after hip and knee arthroplasty, and for the treatment and secondary prophylaxis of VTE. Rivaroxaban is also approved in Europe for secondary prevention of atherothrombotic events after acute coronary syndrome (ACS) with elevated cardiac biomarkers [2–7].

Advantages of NOACs include rapid onset and offset of action and relatively predictable anticoagulation effects [8]. In most patients, routine laboratory monitoring of the anticoagulant effect is not required but the assessment of the estimated renal clearance is necessary [9]. In some cases (e.g., emergencies, bleeding, overdose, and trauma), the anticoagulation status and the alteration of standard laboratory data must be known [10, 11]. An increasing number of patients on long-term treatment with NOACs are encountered in the perioperative setting and it is essential for physicians to be aware of the pharmacological properties of these drugs. The management of those patients requires an involvement of all participating teams (general practitioners, surgeons, anesthesiologists, and other healthcare professionals involved in invasive procedures). Their cessation is indisputable in most elective procedure, but the risk between thrombosis and bleeding should be balanced [12]. In some settings, the therapeutic window is bridged by low molecular weight heparin (LMWH) or unfractionated heparin (UFH) to prevent thromboembolic risk [13, 14]. No specific antidote is currently available in case of bleeding so clinicians have to deal with rescue treatments [15]. The optimal time for NOAC's resumption depends mainly on the postoperative risk of bleeding [16].

This paper aims at providing a review on the management of NOACs in the perioperative setting in accordance with the current literature. This includes the estimation of the bleeding and thrombotic risk of each patient, the period of NOAC's interruption before an invasive procedure, the conditions for heparin bridging during this interruption, the usefulness of common and specific laboratory tests to assess the remaining anticoagulant effect preoperatively, and the time of NOAC's resumption prerequisites for the perioperative management of NOACs. The literature search was performed in PubMed using the following keywords: perioperative, anticoagulant, dabigatran, rivaroxaban, and apixaban. Overall inclusion of papers was limited to studies published until May 30, 2014.

## 2. Indications and Posology of NOACs 

Three molecules are currently available in the clinical setting: dabigatran etexilate (Pradaxa, Boehringer-Ingelheim Pharma GmBH, Ingelheim am Rhein, Germany): 75 mg, 110 mg, and 220 mg capsules, rivaroxaban (Xarelto, Johnson and Johnson/Bayer HealthCare AG, Leverkusen, Germany): 2.5 mg, 10 mg, 15 mg, and 20 mg tablets, and apixaban (Eliquis, Bristol Myers Squibb/Pfizer, Bristol Myers Squibb House, Uxbridge, United Kingdom): 2.5 mg and 5 mg tablets.

Table 1 summarizes the approved indications by the Food and Drug Administration and the European Commission, the posology, and the dose adaptation of the different NOACs.

Table 2 summarizes the main studies leading to the approved indications of NOACs [17–27].

## 3. Clinician Oriented Overview of Pharmacokinetic Properties of NOACs

### 3.1. Dabigatran Etexilate (Pradaxa, Boehringer-Ingelheim Pharma GmBH, Ingelheim am Rhein, Germany): 75 mg, 110 mg, and 220 mg Capsules

Dabigatran etexilate is the prodrug of dabigatran, a selective and reversible oral direct thrombin inhibitor. The plasma peak after ingestion is at 1.5–3.0 hours and the plasma trough level is 11–14 hours after ingestion in healthy volunteers [28]. Bioavailability varies from 3 to 7% depending on the pH encountered in the microenvironment of the gastrointestinal tract. Dabigatran is 35% bound to plasma proteins, allowing theoretically its elimination by hemodialysis. Eighty percent of the drug is directly eliminated in the urine, explaining that, in the setting of renal insufficiency, the anticoagulant effect accumulates. Its elimination half-life rises to 18–24 hours in patients with significantly impaired renal function compared to healthy elderly subjects [29]. Creatinine clearance (CrCl) estimation based on the Cockroft and Gault formula [30, 31] is recommended in elderly patients to calculate doses and avoid overmedication [9]. Twenty percent is conjugated as glucuronides by hepatic metabolism. Dabigatran etexilate is contraindicated in case of severe renal impairment (CrCl < 30 mL/min) in Europe while a lower dose is proposed in North America for CrCl between 30 and 15 mL/min [32]. Hepatic impairment or liver disease with expected impact on survival is also a contraindication [33, 34].

### 3.2. Rivaroxaban

Rivaroxaban is a selective and reversible oral direct FXa inhibitor. The plasma peak after ingestion is at 2–4 hours and the half-life elimination is 5–9 hours in healthy volunteers and 11–13 hours in the elderly [28]. Bioavailability is between 80 and 100% for 10 mg and around 66% for 15 or 20 mg under fasting conditions. Rivaroxaban is bound to plasma proteins in more than 90%, making hemodialysis ineffective to eliminate this drug. About one-third (36%) of the dose is eliminated by renal pathway as unchanged active drug, and the approximately remaining two-thirds of the dose is subject to metabolic degradation. Metabolites are eliminated equally via renal pathway and via hepatobiliary route in the feces [35]. Clearance is mildly influenced by renal function [36]. Rivaroxaban is not recommended in severe renal impairment (ClCr < 15 mL/min) [32].

### 3.3. Apixaban

Apixaban is a selective and reversible oral direct FXa inhibitor. The plasma peak after ingestion is at 2-3 hours and the half-life elimination is 8–15 hours in healthy volunteers [28]. Apixaban is 87% bound to plasma proteins. Bioavailability is around 50%. Apixaban is eliminated via multiple pathways: predominantly via the fecal route (56%) and 25–29% via renal excretion [37]. Apixaban is not recommended in severe renal impairment (ClCr < 15 mL/min) [32].

Table 3 proposes an overview of the main pharmacokinetic properties of direct oral anticoagulants.

## 4. Drugs Interactions 

Two mechanisms are mainly involved in NOACs' metabolism and elimination: the efflux operated by P-glycoprotein (P-gp) and the CYP450 isoform CYP3A4. Dabigatran etexilate is metabolized in dabigatran in the plasma and liver via CYP450-independent mechanisms [38], but DE acts as a substrate for P-gp. Therefore, strong inhibitors or inducers of P-gp may alter the absorption of DE [39]. Rivaroxaban and apixaban are metabolized by CYP3A4/5 and are both substrates for P-gp. Thus, drugs that strongly inhibit or induce CYP3A4 or P-gp or both influence the pharmacokinetic (PK) profile of these NOACs [39, 40].

## 5. Perioperative Management of NOACs

As illustrated in the RE-LY (randomized evaluation of long-term anticoagulation therapy) study [41], 25% of the patients on anticoagulant therapy required a transitory cessation during the two years of follow-up while, in the ROCKET-AF (rivaroxaban once daily oral, direct factor Xa inhibition compared with vitamin K antagonism for prevention of stroke and embolism in atrial fibrillation) study [42], 33% of the patients experienced a temporary interruption of anticoagulant therapy. Forty percent of these interruptions were before surgical or invasive procedure.

In the perioperative context, the balance between thrombosis (in case of anticoagulation interruption) and bleeding (in case of anticoagulation continuation) should be assessed for each patient [43].

### 5.1. The Thromboembolic Risk

#### 5.1.1. For the Arterial Side

In developed countries, atrial fibrillation (AF) affects about 1.5 to 2% of the population. This arrhythmia is associated with a 5-fold increased risk of stroke (AF is associated with 15–20% of all strokes [44]), a 3-fold increased incidence in congestive heart failure, and a higher mortality [45]. Scores that assess thrombotic risk in the perioperative setting are not well established, whereas in certain chronic conditions risks like AF, stratification scores help in decision making when the risk of thrombosis and bleeding must be weighted [46, 47]. The most widely used score is the CHADS_2_ score (congestive heart failure, hypertension, age > 75 years, diabetes, and prior stroke/transient ischemic attack). It is validated for predicting AF-related thromboembolic risk events and helps for the optimal selection of VKAs and NOACs therapies [46, 47]. Since 2010, the CHA_2_DS_2_-VASc score (including cardiovascular disease, atherosclerotic disease, and female sex as additional risk factors) (Table 4) improves the predictive value for thromboembolism. Only a small proportion of patients belong to the low risk and intermediate risk categories [46, 48]. Patients with CHA_2_DS_2_-VASc score ≥ 2 are considered at high risk of thrombosis [46].

The ARISTOTLE (apixaban for reduction in stroke and other thromboembolic events in atrial fibrillation) trial shows a superiority of apixaban over warfarin in terms of stroke or systemic embolism prevention [20, 49] whatever the assessment of stroke risks by CHADS_2_ and CHA_2_DS_2_-VASc [50]. Actually, the assessment of periprocedural thrombotic risk is extrapolated from the risk outside the periprocedural period [12].

#### 5.1.2. For the Venous Side

Patients with venous thromboembolism are at high risks of recurrent thrombosis, thrombus propagation, and embolization until 3 months after diagnosis and initiation of anticoagulation therapy [51]. The risk of recurrence is conditioned by the underlying cause. It decreases if the etiology is provoked (e.g., trauma, fractures, and pregnancy), but if the underlying cause is idiopathic, the risk of recurrence is higher [52].

### 5.2. The Bleeding Risk

The bleeding risk is multifactorial and its assessment needs to consider patient-specific and procedure-specific variables [53].

#### 5.2.1. Patient-Specific Variables

*For the Arterial Side*. HAS-BLED score is used to assess 1-year risk of major bleeding in patients with AF under VKAs [54] (Table 5). Other scores such as ATRIA [55] and HEMORR_2_HAGES [56] scores are also used [57]. All these scores offer a modest predictive performance of the estimation of the bleeding risk in NOACs treated patients with AF. However, HAS-BLED and HEMORR_2_HAGES scores are superior in terms of clinically relevant bleeding for patients on NOACs [58]. Nevertheless, a second analysis of the ARISTOTLE trial shows less bleeding under apixaban compared with warfarin, independently of the HAS-BLED score [50].

*For the Venous Side*. The RIETE score (Registro Informatizado de Enfermedad Tromboembólica) assesses the risk of fatal bleeding in VTE patients and seems better in predicting gastrointestinal than intracranial fatal bleeding [59].

#### 5.2.2. Procedure-Specific Variables

There is little data available to identify the risk of blood loss related to the invasive procedure or surgery [60], except for cardiac surgery [61]. The estimation of bleeding risk for surgery/invasive procedure remains controversial for certain procedures and has low level of evidence [13]. Furthermore, there is a high intercenter variability in red cell blood loss for the main procedure, mainly reflecting differences in surgical techniques. Similarly, classifications into different surgery bleeding risks according to the severity of trauma and the risk of periprocedural bleeding [12, 60, 62–65] are not always easy to use in daily practice. Therefore, it is recommended to develop an institutional guideline and a hospital-wide policy concerning perioperative anticoagulation management in different procedures [63].

### 5.3. Interval between Last Dose and Invasive Procedure or Surgery

The interval between the last dose and the invasive procedure or surgery depends on the bleeding risk of the procedure and the drug half-life (Table 3).

#### 5.3.1. The Invasive Procedure or Surgery

Some procedures defined as minimal procedures with little tissue trauma are at low risk for bleeding [14, 63, 66] and can be achieved without interruption of NOACs (e.g., superficial skin and oral mucosal surgery, wound revisions, nonextraction dental treatment, or cataract surgery). Gastrointestinal endoscopic biopsy without cessation of DE seems to be safe according to current Japanese guidelines [67]. It is recommended to perform the procedure at the trough drug concentration (12 or 24 hours after the last intake) [13, 63, 68].

#### 5.3.2. Drug Metabolism and Elimination

The percentage of drug eliminated after 2, 3, 4, and 5 half-lives is 75%, 87.5%, 93.8%, and 96.9%, respectively [69]. Dabigatran etexilate, rivaroxaban, and apixaban have different drug metabolic and elimination pathways (Table 3). Creatinine clearance (CrCl) must be assessed by the Cockroft and Gault method. Estimation of renal function by Modification of Diet in Renal Disease Equation 4 (MDRD 4) leads to an overestimation of the renal function at lower levels. Thus, many elderly patients with AF would either become incorrectly eligible for NOACs or would receive a too high dose, which may explain the serious incidences of bleeding reported [9, 30].

Some specific populations have an increased half-life of NOACs' elimination and need therefore special attention. This concerns patients with renal or hepatic impairment, particularly the elderly with fluctuating renal function, diuretic use, hypovolemia, liver chronic infections, liver cirrhosis, alcohol abuse, obstructed bile flow, hepatorenal syndrome, and associated coagulopathy. Assessing both renal and liver function must be done preoperatively for every patient on NOACs. The three agents can be used in mild hepatic impairment (Child-Pugh A), while DE and apixaban are allowed in moderate hepatic impairment (Child-Pugh B), but not in severe hepatic impairment (Child-Pugh C) [70].

It is also important to check preoperatively any off-label use or misuse of these NOACs, including the concomitant medications (verapamil, dronedarone, ketoconazole, amiodarone, tacrolimus, etc.), older ages, and extreme body weights. All of these groups should probably require a longer interval of NOACs' arrest, and a measurement of the anticoagulant residual activity should be considered before an invasive procedure.

Several interval schemes based on expert's opinions are proposed taking into account NOACs' pharmacokinetics, patient, and/or type of invasive procedure or surgery (Table 6).

The “*Groupe d'Intérêt en Hémostase Périopératoire*” (GIHP) proposes a unique scheme: 1 day of interruption in case of low risk bleeding surgery or procedure and 5 for other procedures, whatever the molecule, renal function, and concomitant medications [13]. The duration of stopping is proposed empirically to ensure no residual anticoagulant effect in the absence of a validated antidote.

Except for minimal procedures which can be achieved without stopping NOACs, other experts or expert groups propose a window without any NOAC, according to the CrCl and/or the type of surgery. An interval of at least 48 hours (about 3 half-lives) should be maintained for a patient with a CrCl above 50 mL/min to perform surgery or invasive procedure, whatever the molecule. The free interval is increased to at least 4 days for patients on DE with CrCl between 30 and 50 mL/min or for patients on rivaroxaban/apixaban with a CrCl between 15 and 30 mL/min (Table 6). Further large prospective studies are needed to confirm the safety of these perioperative procedures.

### 5.4. To Bridge or Not to Bridge during the Perioperative Interruption

For patients on VKA, the procedure is well established [12, 60]. Guidelines recommend to bridge patients at high risk for thromboembolism (mechanical heart valve, AF, or VTE) with LMWH. For patients at low risk for thromboembolism, they suggest no bridging in case of stopping [60, 71]. Patients classified as being at high risk have more than 10% annual risk for thromboembolism while this risk is reduced to less than 5% for those classified as being at low risk [60].

For VKAs, Siegal et al. [71] as well as Feng et al. [72] showed an increased risk of bleeding with similar thrombotic risk among patients who underwent periprocedural bridging therapy with heparin bridging. Recently, Omran et al. [73] have validated a HAS-BLED score ≥ 3 as the most predictive variable for hemorrhage for patients who had heparin bridging during a perioperative interruption of VKAs before an elective invasive procedure.

Expert's opinions recommend no heparin bridging for NOACs [62, 63, 74], except the GIHP [13]. The last group proposes to stop NOACs 5 days before a surgery with medium or high risk of bleeding, to ensure a complete elimination of the drug in all patients. And if the patient is at high thromboembolic risk (e.g., AF with a history of stroke), they suggest bridging with LMWH or UFH.

Beyer-Westendorf et al. [14] had recently presented the first prospective data from a national registry that supported the concept of short-term interruption without heparin bridging.

They concluded that if a surgery or an invasive procedure requires a NOAC's arrest, most patients can safely interrupt NOACs for a short period without heparin bridging. In case of heparin bridging therapy, the rate of cardiovascular events is not reduced. There is a significantly higher rate of bleeding complications due to heparin bridging or major procedures. However, patients at cardiovascular risk undergoing major procedures may benefit from heparin bridging because their outcome in terms of cardiovascular risk is increased and because, in this particular setting, heparin bridging is not an independent factor for bleeding risk.

Those data do not support a systematic bridging therapy but highlight its probable benefit in patients at cardiovascular risk undergoing high risk surgery.  Table 7 shows categories of procedures defined by the severity of tissue trauma and the risk for periprocedural bleeding [14].

During the ROCKET-AF trial, patients who required temporary interruption of anticoagulant therapy for surgery and invasive procedure were bridged only in 6% of the cases, predominantly by LMWH. The rate of major bleeding was similar in bridged and nonbridged patients. The incidence of bleeding (major and nonmajor clinically relevant bleeding according to International Society on Thrombosis and Hemostasis definitions [75]) was higher in case of bridging therapy, while stroke and systemic embolism were similar in both groups [42]. The study suffers several biases such as the use of bridging therapy in a nonrandomized way and the too low number of events.

Devices implants in most of Canadian centers are performed with NOAC interruption without LMWH bridging [76]. Again, this study demonstrates the necessity of an individual assessment of each patient, on a case-by-case basis [77].

Thus, there is no universal strategy for periprocedural management of NOACs, but a stepwise approach can be considered. Some prerequisites are essential to allow periprocedural decision (Table 8).

The decision about perioperative NOACs management should be written in the medical record.

### 5.5. A Particular Case: Anesthesia Procedures

#### 5.5.1. Neuraxial Anesthesia

For patients without thrombotic risk (assessed by CHA_2_D_2_-VASc score), Benzon et al. [78] recommend 5 half-life intervals between NOAC's discontinuation and a neuraxial puncture (with or without epidural catheter placement). For patients with high risk of stroke or VTE, this interval can be shortened to 2 or 3 half-lives, or it can stay at 5 half-lives if LMWH bridging is associated. Llau and Ferrandis [79] provided recommendations based on NOAC's pharmacokinetics in the setting of thromboprophylaxis. For spinal anesthesia, if the puncture is atraumatic, the first dose of DE can be administrated 1–4 hours after the end of surgery and after 6–8 hours for rivaroxaban. If the puncture is traumatic or hemorrhagic, the first dose of DE or rivaroxaban is delayed by 24 hours. For epidural anesthesia, DE cannot be administrated if a permanent catheter is inserted. For rivaroxaban, the first dose after epidural anesthesia is administered 6–10 hours after the end of surgery. An interval of 18 hours is recommended before the removal of the epidural catheter (22–26 hours for elderly patients) and at least 4 hours after removal. The European Society of Anaesthesiology (ESA) [80] recommends extreme caution when using neuraxial blockade in the presence of rivaroxaban/apixaban. For dabigatran, the manufacturer advises against its use in the presence of neuraxial blockade. Because of a potential risk of retroperitoneal hematoma in lumbar plexus and paravertebral blocks, ESA recommends the same guidelines for these two peripheral nerve blocks as for neuraxial blockades [78, 80].

#### 5.5.2. Other Peripheral Nerve Blocks

ESA does not routinely apply the same guidelines as for neuraxial blockades [81], but the American Society of Regional Anesthesia (ASRA) does [78].

### 5.6. A Particular Case: Atrial Fibrillation Ablation

Uninterrupted warfarin therapy with therapeutic anticoagulation has been shown to be associated with lower risk of periprocedural thromboembolic events after AF ablation and is increasingly being accepted as the preferred anticoagulation strategy [82]. However, increasingly more patients are being treated with NOACs, thereby complicating the periprocedural anticoagulation management.

For dabigatran, there is no consensus regarding the management of patients taking dabigatran who are referred for AF ablation. The results depend on the period of dabigatran interruption. Nearly uninterrupted anticoagulation (holding the morning dose), which theoretically has a better protection from periprocedural thromboembolism, was associated with both increased bleeding and thromboembolic events, especially in the nonparoxysmal AF [83]. Other observational studies with a more interrupted approach showed equivalent bleeding and embolic events compared to therapeutic warfarin [84–87].

For rivaroxaban, uninterrupted rivaroxaban appears to be a feasible and safe alternative to uninterrupted warfarin therapy in patients undergoing AF ablation. Future larger and randomized trials are needed to confirm those preliminary data [83].

## 6. Management in an Emergency Situation 

In case of a surgery or invasive procedure, the degree of emergency should be assessed in order to decrease the bleeding risk: NOACs must be discontinued, timing of the last dose intake should be known, and if possible, invasive procedure should be delayed until the NOACs reach trough concentration. Waiting and postponing semiurgent procedures may be a reasonable strategy to prevent bleeding. In case of bleeding emergencies under NOACs, several difficulties are encountered: there is currently no antidote and no rapid quantitative measurement of the anticoagulant effect (see laboratory testing for NOACs in a perioperative setting), strategies to reverse the anticoagulant effect are poor, and, depending on the residual concentration of NOACs, administration of factors is rendered ineffective [15, 63].

It is very important to establish a hospital-wide policy for bleeding management in patients taking NOACs, based on the available blood products and laboratory assays in each institution. The procedure must be easily accessible (e.g., intranet site, personal digital assistant (PDA)). Some workgroups propose algorithms based on NOAC's plasma concentration, but their quantitative measurements are not currently routinely performed [88].

In case of bleeding that does not respond to supportive measures (surgical hemostasis, embolization, fluid replacement, etc.) in patients taking DE, ensure adequate diuresis and suggest hemodialysis. Dabigatran is 35% bound to plasma proteins, theoretically allowing its elimination by hemodialysis, but clinical experience is limited [89–91]. However, hemodialysis might be more effective than unspecific reversal treatment with factor concentrates. A single-center study including patients with end-stage renal disease measured DE elimination with hemodialysis. Four hours of hemodialysis with 200 mL/min and 400 mL/min targeted blood flow removed 48.8% and 59.3% of total dabigatran from the central compartment, respectively. There was a linear relation between anticoagulant activity of dabigatran and its plasma levels. Minor redistribution of dabigatran (<16%) after the end of the hemodialysis session occurred. These results support hemodialysis as a suitable approach to eliminate dabigatran in emergency situations [92]. An extracorporeal renal replacement therapy filter can also be easily added during emergent cardiac surgery [93]. However, a special care to bleeding at the punctures sites is necessary and therefore ultrasound guided techniques are very useful. Hemoperfusion over a charcoal filter is under evaluation [89]. Oral activated charcoal may be effective only in case of a recent ingestion within 2 hours [89].

An analysis of major bleeding patients in the RE-LY study concludes that the overall resources required to manage bleeding were not greater than those after warfarin-related bleeding. For patients treated with DE, red cells transfusions were higher, plasma transfusions were lower, the stay in intensive care unit was shorter, and there was a lower trend in mortality compared with patients treated with warfarin. Based on these results, they concluded on a safety profile of DE [94].

There is currently no specific hemostatic agent for the reversal of NOACs in case of bleeding or in emergency situations but different antidotes are under development. Andexanet alfa (PRT064445) is a truncated form of enzymatically inactive FXa: it reverses dose-dependently the inhibitory activity and corrects* ex vivo* clotting times [95]. For dabigatran, a humanized selective and specific monoclonal antibody fragment (idarucizumab) is under development [96]. Aripazine (PER977), another small synthetic molecule, reverses anticoagulant activity of all clinically used NOACs in rat bleeding models [97].

Hemostatic agents used for life-threatening bleeding are shown in Table 9.

## 7. Laboratory Testing for NOACs in a Perioperative Setting

NOACs do not require routine coagulation monitoring. This point is considered as an advantage for the physicians and an improvement of quality of life for their patients. However, point measurement of NOACs may be required in several clinical situations including the perioperative setting [8, 10, 11, 78, 98, 99].

For VKAs, it is well established that, at time of surgery (INR on the day before surgery), an elevated INR (i.e., ≥2) will increase the risk of bleeding and a near normal or normal INR (i.e., ≤1.5) will not [60]. But, for NOACs, the residual drug level that can be considered as safe is not known, except for dabigatran. The residual dabigatran concentration that is recommended before special intervention (such as surgery) is provided in the Committee for Medicinal Products for Human Use (CHMP) assessment report from the European Medicine Agency that states “(…)* dabigatran concentration under 48 ng/mL is equivalent to at least 75% of dabigatran's elimination and should be recommended*” [100]. A French group of experts called GIHP proposed the threshold of 30 ng/mL (for dabigatran and rivaroxaban) [88].

Details of these recommendations are presented in Table 10 [88].

### 7.1. Which Laboratory Tests Should We Use in the Perioperative Setting and How to Interpret Them?

Activated partial thromboplastin time (aPTT) and prothrombin time (PT) are global assays not reflecting plasma NOACs concentrations. They are not suitable for precise quantification of the anticoagulant effect. Thrombin time (TT) was demonstrated to be too sensitive towards dabigatran [89, 101]. However, this may guide the clinician in the perioperative setting since a normal TT indicates no clinically relevant anticoagulant effect of dabigatran. The strong sensitivity of TT towards dabigatran leads to the development of a calibrated diluted thrombin time (dTT) with dabigatran standards to calculate the dabigatran plasma concentration. Several studies showed that the dTT or the ecarin chromogenic assay (ECA) highly correlates with dabigatran plasma concentrations measured by LC-MS/MS in patient's plasma [102–104]. However, one limitation of dTT is that, in case of switching therapy (i.e., from heparins/heparinoids to dabigatran etexilate or from hirudin and derivatives to dabigatran etexilate), they will be slightly influenced by the presence of such inhibitors in the plasma. This implies the necessity of an accurate anamnesis of the drugs taken by the patient to avoid overestimation of drug concentrations in plasma. In addition, for the accurate determination of dabigatran plasma concentrations below 50 ng/mL, the more sensitive liquid chromatography-mass spectrometry/mass spectrometry (LC-MS/MS) method is still required [102, 104].

For rivaroxaban, chromogenic anti-Xa assay has been proven to accurately estimate the plasma rivaroxaban concentrations > 30 ng/mL [105]. Due to their good sensitivity towards the inhibition of FXa by apixaban, chromogenic anti-Xa assays calibrated with specific apixaban calibrators should be performed to estimate plasma drug concentration [106–108]. However, taking into account the lower sensitivity of chromogenic assays compared to LC-MS/MS and the variability of coagulation analysers that may further increase the imprecision at the lowest concentrations, detection and quantitation of lower levels (<30 ng/mL for rivaroxaban and <15 ng/mL for apixaban) still require LC-MS/MS analyses [108, 109].

Even if these specific coagulation tests to assess NOACs are promising, they suffer from difficulties to be implemented easily in the clinical setting. Moreover, assessment of lower levels of NOACs encountered in the perioperative setting is challenging due to the limit of quantitation of these tests but improvements in the low range are under development by several companies.

In addition, to correctly interpret the results of laboratory assay, some information needs to be collected: drug, indication, and the timing of the last dose administration (due to the short half-life of NOACs) [110]. The interpretation of the results requires education of the front staff in many specialties [98]. Finally, it has been clearly shown that NOACs influence the results of different coagulation assays used in the perioperative setting leading to misinterpretations [41] (Table 11).

### 7.2. A Particular Case: Atrial Fibrillation Ablation

The ideal management of oral anticoagulation during catheter ablation for AF 112–114 is still controversial with a wide range of procedures available. During AF ablation, it is now recommended to achieve and maintain an ACT of 300 to 400 seconds in order to reduce the risk of systemic thromboembolism [111]. However, the ACT is affected by a lot of preanalytical [112] and analytical variables [113, 114]. Finally, target ACT should be redetermined for the periprocedural use of NOACs for AF ablation [111–117].

## 8. Resumption of the NOACs after Invasive Procedure or Surgery

Once again, in the postoperative period, the bleeding risk must be weighted with the thromboembolic risk; however the risk for major bleeding exceeds the risk for thromboembolic complications after surgery [118]. Regarding the thromboembolic risk, Kaatz et al. [119] concluded that patients with chronic AF had twice as much risks of postoperative stroke, especially in neurosurgery and vascular surgery. The bleeding risk in the postoperative period is mainly due to patient's characteristics (bridging therapy, mitral mechanic heart valve, active cancer, prior bleeding history, and reinitiation of heparin therapy within 24 hours after surgery), even if a premature heparin resumption is an avoidable risk factor [120]. But first of all surgical bleeding risk must be under control [121]. For NOACs, given the fact that full anticoagulation occurs between 2 and 4 hours (Table 3) and that no antidote is available, resumption of DE, rivaroxaban, and apixaban should be performed at least 48 hours after the high risk procedures [12]. This delay can expose patients at risk for thromboembolism. Twenty-four hours are recommended before resuming oral anticoagulation after a procedure at low risk of bleeding [60, 62]. Other schemes exist in order to minimize bleeding risks: consider a stepwise resumption of NOACs [16, 77] or administer prophylactic doses of LMWH early after the surgery, and restart full doses of NOACs only after 3 or 4 days [122]. Heparin bridging can be useful if the patient cannot tolerate oral medication (e.g., in ileus or postoperative nausea and vomiting) [66]. In the postoperative setting, it is essential to reassess patient's renal function, especially for elderly who are subject to dehydration and for patients taking DE. For LMWH, doses must be adapted in case of extreme body weight (body mass index above 30 kg/m^²^) and poor renal function. If CrCl is less than 30 mL/min, the use of unfractionated heparin is more indicated [12, 123].

## 9. Conclusions

In the field of oral anticoagulation, clinicians will be more frequently confronted about their management of NOACs in the pre- and postoperative setting. Prerequisites are essential for NOAC's pharmacokinetics, indications, drug interactions, and alterations of standard laboratory assays. Actual data are based mainly on expert's opinions, except one national registry study. In some situation, interruption periods of NOACs are necessary and should be based on their respective half-life, on the bleeding risk of procedures, and on the thromboembolic risk of patients. Some scores such as CHA_2_DS_2_-VASc and HAS-BLED should help clinicians in their decisions. Given their shorter half-life, no heparin bridging during the interruption interval seems necessary, except for patients at high cardiovascular risk undergoing major surgery.

Further clinical trials over perioperative management of patients under NOACs are still required. Emergency surgeries, invasive procedures, or bleeding patients remain a real challenge given the absence of antidote. Possibilities of reversal are poor and based on few experimental data and case reports. Furthermore, rapid quantitative measurements of the anticoagulant effect are not available in most institutions. Awaiting future clinical trial data, it seems to be important to establish a hospital-wide policy for bleeding management in patients taking NOACs, in accordance to the available blood products and laboratory assays of each institution.

## Figures and Tables

**Table 1:** Summary of approved indications, posology and dose adaptation of the different NOACs.

	Dabigatran etexilate (Pradaxa)	Rivaroxaban (Xarelto)	Apixaban (Eliquis)
VTE Prophylaxis	(i) 220 mg/day (2 capsules of 110 mg OD) or (ii) 150 mg/day (2 capsules of 75 mg OD) if CrCl 30–50 mL/min, if >75 years, if verapamil, amiodarone and quinidine THR: 28–35 days TKR: 10 days	10 mg/day (1 tablet of 10 mg OD) THR: 5 weeks TKR: 2 weeks	5 mg/day (1 tablet of 2.5 mg BID) THR: 32–38 days TKR: 10 days

Non-valvular atrial fibrillation	(i) 300 mg/day (1 capsule of 150 mg BID) (ii) 220 mg/day (EU) (1 capsule 110 mg BID) (a) if >80 y or verapamil 150 mg/day (US) (1 capsule of 75 mg BID) (b) if CrCl between 15–30 mL/min (c) if dronedarone/ketoconazole (US)	(i) 20 mg/day (1 tablet of 20 mg OD) (ii) 15 mg/day (1 tablet of 15 mg OD) if CrCl between 15–49 mL/min	(i) 10 mg/day (1 tablet of 5 mg BID) (ii) 5 mg/day (1 tablet of 2.5 mg BID) if at least 2 of the following conditions: ≥80 years, ≤60 kg or serum creatinine ≥1.5 mg/dL; or if CrCl 15–29 mL/min

VTE treatment and secondary prophylaxis	(i) 150 mg BID after 5–10 days parenteral anticoagulation (ii) 1 capsule 75 mg BID if CrCl <30 mL/min (US) (iii) Adopted indication CHMP° (april 2014) (EU)	(i) Treatment phase: 30 mg/day (1 tablet of 15 mg BID) for 21 days (ii) Secondary prevention: 20 mg/day (1 tablet of 20 mg OD) 15 mg/day (1 tablet of 15 mg OD) if CrCl between 15–49 mL/min and the risk of bleeding outweighs the risk of recurrent DVT or PE	*✗*

Prevention of atherothrombotic events after ACS with elevated cardiac biomarkers	*✗*	5 mg/day (1 tablet of 2.5 mg BID) in association with ASA (75–100 mg) alone or ASA + clopidogrel (75 mg)	*✗*

*✗*: Off-label; BID: twice daily; CrCl: creatinine clearance; VTE: venous thromboembolism; OD: once daily; PE: pulmonary embolism; THR: total hip replacement TKR: total knee replacement; °Committee for Medicinal Products for Human Use (CHMP), ASA: acetylsalicylic acid.

**Table 2:** Summary of the main studies leading to approved indications of NOACs.

Clinical context	NOAC	Other anticoagulant	Conclusion (NOACs versus other drugs)
VTE^0^ prophylaxis after orthopedic surgery			
RE-MODEL	Dabigatran etexilate 150 or 220 mg OD	Enoxaparin 40 mg OD SC^5^	Same efficacy and safety profile after TKR^1^
RE-NOVATE II	Dabigatran etexilate 220 mg OD	Enoxaparin 40 mg OD SC	Same profile in term of safety and bleeding after THR^2^
RECORD	Rivaroxaban 10 mg OD	Enoxaparin 40 mg OD SC	More effective, without increasing major bleeding after THR/TKR
ADVANCE II	Apixaban 2.5 mg BID	Enoxaparin 40 mg OD SC	More effective without increased bleeding after TKR
ADVANCE III	Apixaban 2.5 mg BID	Enoxaparin 40 mg OD SC	Lower rate of VTE without increased bleeding after THR
Non-valvular atrial fibrillation			
RE-LY^3^	Dabigatran etexilate 110 mg or 150 mg BID	Adjusted dose warfarin (INR 2-3)	Efficacy superior for the prevention of stoke with a similar rate of major bleeding
ROCKET-AF^4^	Rivaroxaban daily dose 20 mg	Adjusted dose warfarin	Non-inferiority, no significant difference in term of bleeding
ARISTOTLE	Apixaban 5 mg BID	Adjusted dose warfarin (INR 2-3)	Superior in preventing stroke or systemic embolism, less bleeding and lower mortality
VTE Treatment			
RE-COVER II	Dabigatran etexilate 150 mg BID after 5–11 days of LMWH^6^ or UFH^7^	Warfarin	Similar effect on VTE recurrence, lower risk of bleeding for the treatment of acute VTE
EINSTEIN	Rivaroxaban 15 mg BID for 3 weeks following by 20 mg OD	Enoxaparin SC following by vitamin K antagonist	Simple, single drug approach. Improve benefit-to-risk of anticoagulation
Acute coronary syndrome			
ATLAS ACS	Rivaroxaban 2.5 BID	Placebo	Reduced the composite endpoint of death from cardiovascular causes, myocardial infarction or stroke. No increase of fatal bleeding.

^0^VTE: venous thromboembolism, ^1^TKR: total knee replacement, ^2^THR: total hip replacement, ^3^RE-LY: Randomized Evaluation of Long-Term Anticoagulation therapy, ^4^ROCKET-AF: Rivaroxaban Once Daily Oral, Direct factor Xa Inhibition Compared with Vitamin K antagonism for Prevention of Stroke and Embolism in Atrial Fibrillation, ^5^SC: Subcutaneously, ^6^LMWH: low molecular weight heparin, ^7^UFH: unfractionated heparin.

**Table 3:** Overview of main pharmacokinetic properties of NOACs.

	Dabigatran etexilate	Rivaroxaban	Apixaban
Plasma peak (hours)	1.5–3.0	2.0–4.0	3.0-4.0

Elimination half-life (hours)	11–14: healthy volunteers 18–24: significantly impaired renal function	5–9: healthy volunteers 11–13: elderly	8–15: healthy volunteers

Protein binding (%)	35%	>90%	87%

Elimination (%)	80% active renal 20% faecal	33% non-active renal 66% metabolized: (metabolism: 50% renal and other half by hepatobiliary route)	Multiples pathways: 25%–29% renal 56% by faecal route

Bioavailability	3–7% PH sensitive	80–100% 10 mg 66%: 15–20 mg under fasting conditions	±50%

**Table 4:** CHA_2_DS_2_-VASc Score.

Acronym	CHA_2_DS_2_-Vasc Score	Points
C	Congestive heart failure	1
H	Hypertension	1
A_2_	Age ≥ 75 years	2
D	Diabetes mellitus	1
S_2_	Stroke, transient attack, or thromboembolism	2
V	Vascular disease (prior myocardial infarction, peripheral arterial disease, aortic plaque)	1
A	Age 65–74 years	1
Sc	Sex category: female	1

**Table 5:** HAS-BLED score.

HAS-Bled Score	Risk Factor	Points
H	Hypertension (uncontrolled, systolic blood pressure >160 mmHg)	1
A	Abnormal renal function or liver function	1 or 2 (each 1)
S	Stroke	1
B	Bleeding history or predisposition to bleeding (e.g., bleeding diathesis, anemia)	1
L	Labile INR	1
E	Elderly (age > 65 years)	1
D	Drug (antiplatelet, nonsteroïdal anti-inflammatory drugs) or alcohol abuse	1 or 2 (each 1)

**Table 6:** Schemes of discontinuation of NOACs.

	Dabigatran	Rivaroxaban	Apixaban
Baron et al. [12]	CrCl ≥ 50 mL/min: 1 or 2 days CrCl < 50 mL/min: 3–5 days	≥1 day if CrCl normal CrCl 60–90 mL/min: 2 days CrCl 30–59 mL/min: 3 days CrCl 15–29 mL/min: 4 days	CrCl > 60 mL/min: 1 or 2 days CrCl 50–59 mL/min: 3 days CrCl 30–49 mL/min: 5 days

Liew et al. [64]	No/minimal residual effect at surgery (4-5 drug half-lives) CrCl > 50 mL/min: 3 days CrCl 30–50 mL/min: 4 to 5 days Mild-moderate effect at surgery (2-3 drug half-lives) CrCl > 50 mL/min: 2 days CrCl 30–50 mL/min: 3 days	No/minimal residual effect at surgery (4-5 drug half-lives) CrCl > 50 mL/min: 3 days CrCl 30–50 mL/min: 4 to 5 days Mild-moderate effect at surgery (2-3 drug half-lives) CrCl > 50 mL/min: 2 days CrCl 30–50 mL/min: 3 days	No/minimal residual effect at surgery (4-5 drug half-lives) CrCl > 50 mL/min: 3 days CrCl 30–50mL/min: 4 to 5 days Mild-moderate effect at surgery (2-3 drug half-lives) CrCl > 50 mL/min: 2 days CrCl 30–50 mL/min: 3 days

Kozek-Langenecker [93]	2 days-interval may be sufficient for low-risk intervention 4 days at least for high risk intervention and/or CrCl < 50 mL/min	1 day for low-bleeding interventions 2 days in high bleeding risk interventions	1 day for low-bleeding interventions 2 days in high bleeding risk interventions

Spyropoulos and Douketis [62]	CrCl > 50 mL/min: Low risk bleeding surgery°: last dose 2 days before surgery High risk bleeding surgery*: Last dose 3 days before surgery CrCl 30–50 mL/min: Low risk bleeding surgery°: Last dose 3 days before surgery High risk bleeding surgery*: Last dose 4-5 days before surgery	CrCl > 30 mL/min: Low risk bleeding surgery°: last dose 2 days before surgery High risk bleeding surgery*: Last dose 3 days before surgery CrCl 15–29.9 mL/min: Low risk bleeding surgery°: Last dose 3 days before surgery High risk bleeding surgery*: Last dose 4 days before surgery	CrCl > 50 mL/min: Low risk bleeding surgery°: last dose 2 days before surgery High risk bleeding surgery*: Last dose 3 days before surgery CrCl 30–50 mL/min: Low risk bleeding surgery°: Last dose 3 days before surgery High risk bleeding surgery*: Last dose 4 days before surgery

Sié et al. [13]	Low risk procedure^1^: 1 day Other procedures than low risk bleeding procedures^2^: stop 5 days before surgery/invasive procedures	Low risk procedure^1^: 1 day Other procedures than low risk bleeding procedures^2^: stop 5 days before surgery/invasive procedures	Low risk procedure^1^: 1 day Other procedures than low risk bleeding procedures^2^: stop 5 days before surgery/invasive procedures

°Low-risk bleeding surgery: 2-day risk of major bleed 0%–2%; *High-risk bleeding surgery: 2%–4%.

^1^Low risk procedure: in case of bleeding, if it occurs, will be low abundance, non-critical in its location and/or easily controlled by simple mechanical hemostasis; ^2^High risk procedure: the probability of significant bleeding cannot be excluded or, any surgery that is usually hemorrhagic or for which the risk of bleeding would be unacceptable.

**Table 7:** Categories of procedures according to the severity of tissue trauma and the risk for peri-procedural bleeding.

Minor procedures with little tissue trauma	
Superficial skin and oral mucosal surgery, skin biopsies Wound revisions Non-extraction dental treatment	

Minor procedures with little tissue trauma, but relevant bleeding risk	

Transluminal cardiac, arterial, and venous interventions Pacemaker-related surgery Pleura and ascites punctures Cataract surgery Arthroscopy, endoscopy, laparoscopy Organ biopsies Dental extraction Hernia repair Intramuscular and paravertebral injections	

Major procedures with relevant tissue trauma and high bleeding risk	

Open pelvic, abdominal and thoracic surgery Brain surgery Major orthopedic and trauma surgery Vascular surgery	

**Table 8:** Essential prerequisites allowing perioperative decisions.

Molecule	Type
	Dose
	Indication

Patient	Thromboembolic risk—bleeding risk
	Renal function (CrCl—Cockroft and Gault formula)
	Hepatic function
	Concomitant drugs
	Approved NOAC's indication

Procedure	Type and technique
	Bleeding risk
	Date of its achievement (Day 0)

Anesthesia	General and/or regional (neuroaxial or peripheral nerve blocks)

**Table 9:** Coagulation factor and pro-hemostatic agents.

Concentrate of factors (II, (VII), IX et X): prothrombin complex concentrate, PCC, PPSB (Cofact, Confidex, Octaplex, Beriplex)	25 U/kg, once or two times*

Concentrate of activated factors: idem PCC + VIIa, FEIBA, (FEIBA)	50 IE/kg, max 200 IE/kg/day*

Factor VIIa: (Novoseven)	Needs further evaluation: 90 *μ*g/kg

Antifibrinolytics (Tranexamic acid (Exacyl), Aminocaproïc acid), Desmopressin (Minirin)	No clinical data in this context

Fresh Frozen Plasma (FFP)	Not useful to reverse anticoagulation, expand plasma volume in case of massive transfusion

Protamin, vitamin K	No effect in case of NOACs bleeding

*PCC or aPCC utilization is based of few experimental data, can be considered if immediate hemostatic support is essential in case of life-threating bleeding (need for ≥4 red cells transfusions and exogenous cathecolamins for hemodynamic stabilization).

**Table 10:** Perioperative management of NOACs (dabigatran and rivaroxaban)—Proposal for recommendations from the GIHP (“Groupe d'Intérêt en Hémostase Périopératoire”).

Measured concentration	Recommendations
<30 ng/mL	Operate

30–200 ng/mL	Wait up to 12 h and obtain new dosage or (if time is not compatible with emergency) Operate, if abnormal bleeding: antagonise the anticoagulant effect

200–400 ng/mL	Wait up to 12 h and obtain new dosage or (if time is not compatible with emergency) Maximise delay surgery Discuss hemodialysis, especially if CrCl < 50 mL/min (with dabigatran only) Operate, if abnormal bleeding: antagonise

>400 ng/mL	Overdose-Major haemorrhagic risk Discuss haemodialysis before surgery (with dabigatran only)

**Table 11:** Influence of dabigatran, rivaroxaban and apixaban on coagulation tests used in the perioperative setting.

	Dabigatran	Rivaroxaban	Apixaban
Prothrombin Time (PT)	Time prolonged + (relative to reagent sensitivity)	Time prolonged + to +++ (relative to reagent sensitivity)	Time non-prolonged or prolonged + (relative to reagent sensitivity)

Activated Partial Thromboplastin Time (aPTT)	Time prolonged + to +++ (relative to reagent sensitivity)	Time prolonged + (relative to reagent sensitivity)	Time prolonged + (relative to reagent sensitivity)

PT-based coagulation factors measurement (II, VII, IX, X)	Slightly decreased (depending on the reagent)	Slightly decreased (depending on the reagent)	Slightly decreased (depending on the reagent)

APTT-based coagulation factors measurement (VIII, IX, XI)	Slightly decreased (depending on the reagent)	Slightly decreased (depending on the reagent)	Slightly decreased (depending on the reagent)

Fibrinogen	No influence or slightly decrease (depending on the reagent)	No influence	No influence

Thrombin Time	Time prolonged +++++	No influence	No influence

Anti-Xa based antithrombin measurement	No influence	Increased: 10% per 100 ng/mL	Increased: 10% per 100 ng/mL

Anti-IIa based antithrombin measurement	Increased: 5–10% per 100 ng/mL	No influence	No influence
